# Practical Ranges of Loudness Levels of Various Types of Environmental Noise, Including Traffic Noise, Aircraft Noise, and Industrial Noise

**DOI:** 10.3390/ijerph8061847

**Published:** 2011-05-26

**Authors:** Erik M. Salomons, Sabine A. Janssen

**Affiliations:** Urban Environment, TNO, Van Mourik Broekmanweg 6, 2628 XE Delft, The Netherlands; E-Mail: sabine.janssen@tno.nl

**Keywords:** environmental noise policy, loudness level, noise-related health effects

## Abstract

In environmental noise control one commonly employs the A-weighted sound level as an approximate measure of the effect of noise on people. A measure that is more closely related to direct human perception of noise is the loudness level. At constant A-weighted sound level, the loudness level of a noise signal varies considerably with the shape of the frequency spectrum of the noise signal. In particular the bandwidth of the spectrum has a large effect on the loudness level, due to the effect of critical bands in the human hearing system. The low-frequency content of the spectrum also has an effect on the loudness level. In this note the relation between loudness level and A-weighted sound level is analyzed for various environmental noise spectra, including spectra of traffic noise, aircraft noise, and industrial noise. From loudness levels calculated for these environmental noise spectra, diagrams are constructed that show the relation between loudness level, A-weighted sound level, and shape of the spectrum. The diagrams show that the upper limits of the loudness level for broadband environmental noise spectra are about 20 to 40 phon higher than the lower limits for narrowband spectra, which correspond to the loudness levels of pure tones. The diagrams are useful for assessing limitations and potential improvements of environmental noise control methods and policy based on A-weighted sound levels.

## 1. Introduction

Environmental noise has serious effects on the health of people. Health effects considered by the World Health Organization include annoyance, sleep disturbance, and cardiovascular disease [1,2]. Exposure-response relations for these end points indicate that the prevalence of noise-related health effects gradually increases with increasing noise exposure [1,3,4].

Noise exposure for the exposure-response relations is represented by an A-weighed sound level (averaged over the day, evening, and night periods). The A-weighted sound level is related to health effects of environmental noise, but is only a limited representation of direct perception of noise. A quantity that is more closely related to the direct perception of noise is the loudness level. However, while annoyance caused by environmental noise is related to the loudness of the noise [5], it also depends on other acoustic and personal factors. Similarly, sleep disturbance by environmental noise may be related to the loudness of the noise but also depends on other factors.

The A-weighting curve for rating noise was originally derived from an equal-loudness contour for pure tones, the 40 phon Fletcher-Munson curve [6], so equal A-weighted sound levels would approximately correspond to equal loudness. There have been several revisions of the equal-loudness contours for pure tones [7–9]. Further, calculation methods have been developed for broadband noise (see Section 2), which show that the shape of the spectrum has a large effect on the loudness. If one considers loudness level variations at constant A-weighted sound level it becomes clear that the A-weighted sound level is only a limited representation of human noise perception. For example, several authors have reported that A-weighting underestimates loudness at low frequency (LF), and therefore would be inappropriate for LF environmental noise control [10–13]. Various other studies on annoyance caused by LF noise have been reported [14–20], but a clear picture about the relation between loudness and annoyance by LF noise does not emerge from these studies. A complicating factor is that annoyance depends also on other acoustic characteristics than loudness. For example, tonal noise is found to be more annoying than broadband noise at the same sound level [21–23]. In particular LF noise often has tonal components (for example, noise from 50 Hz transformers [24]), so tonality and LF character may interfere [25].

For assessing limitations and potential improvements of current noise control methods based on the A-weighted sound level, it would be useful to have a global picture of the ranges of loudness levels that occur in practice, for various types of environmental noise such as traffic noise, aircraft noise, and industrial noise. A relevant question is: how much does the loudness level vary in practice at constant A-weighted sound level? The analysis presented in this note provides an answer to this question. It should be noted that we do *not* suggest that the loudness level is a better indicator of environmental noise than the A-weighted sound level is, as ‘corrected’ A-weighted levels may be just as good as loudness levels.

A distinction should be made between outdoor levels and indoor levels. The exposure-response relations for the health end-points of environmental noise are based on outdoor levels, measured or calculated at the most-exposed façade of a dwelling. The façade levels are an approximation for the ‘true’ noise exposure of people. A possible improvement of this approximation is to take into account also indoor levels, since noise-related health effects may originate to a large extent from indoor noise levels. For sleep disturbance indoor levels in bedrooms play a role. Therefore, this note also considers indoor levels, calculated from outdoor levels and a typical spectrum of façade insulation. However, the effect of façade insulation on environmental noise annoyance is a complex issue, since various non-acoustic factors also play a role, such as expectations of people with respect to the insulation, and the opening and closing of windows. A further complication of indoor levels is the spatial variations in LF sound fields in rooms, with pronounced regions of high and low sound levels due to standing waves [26].

The article is organized as follows. Section 2 describes standard methods for calculating loudness, both loudness of pure tones and loudness of broadband noise spectra. Section 3 presents the analysis of loudness levels calculated for various spectra of environmental noise, including pure tones in Section 3.2, band-limited noise in Section 3.3, and broadband noise representative of traffic noise, aircraft noise, and industrial noise in Section 3.4. Conclusions are presented in Section 4.

## 2. Calculation Methods

The international standard ISO 226:2003 [8], which is an updated version of ISO 226:1987 [7], presents a mathematical relation between the loudness level, in phon, the frequency, and the sound pressure level of pure tones. The relation defines equal-loudness contours as a function of frequency and sound pressure level, covering the frequency range from 20 to 12,500 Hz. The relation applies to binaural perception by persons between 18 and 25 years, with normal hearing, under free field listening conditions. The human hearing threshold as a function of frequency is also specified by the two versions of the standard. The ISO 226:2003 standard is based on an extensive set of experimental loudness studies performed in various countries [9]. The equal-loudness contours according to ISO 226:2003 deviate from the contours according to the (withdrawn) standard ISO 226:1987, which is based on older measurements [27].

Environmental noise usually consists of sound signals with a broad spectrum, covering both the low frequency region, say between 20 Hz and 200 Hz (various definitions of the LF region exist), and the higher frequency region up to about 5 or 10 kHz. The A-weighted spectrum of noise emitted by road vehicles (passenger cars, trucks) has the highest levels in the frequency bands around 1 kHz. With increasing distance from the source, however, the spectrum gets a stronger LF signature, due to frequency-dependent air absorption and screening attenuation by obstacles such as noise barriers and buildings [28–31]. A notorious example of LF noise is the noise generated by an aircraft during the start of the take-off roll on the runway [12,32,33]. This type of aircraft noise is sometimes referred to as aircraft groundnoise. Various industrial noise sources also generate noise spectra with strong LF components.

The calculation or prediction of the loudness of a noise signal with a broad spectrum is a complex problem. The theory of psychoacoustics describes the human hearing system as a set of auditory band-pass filters, corresponding to a set of contiguous frequency bands that are called critical bands [34]. Each critical band corresponds to a separate area on the basilar membrane in the inner ear. Critical bands are related to auditory masking: two tones within a critical band are perceived as a single sound while two tones that are not within the same critical band are perceived as two separate tones with a larger total loudness. Loudness of broadband noise also depends on the critical bands. If the bandwidth of band-limited noise is gradually increased, loudness stays about the same for bandwidths smaller than the critical bandwidth and increases for bandwidths larger than the critical bandwidth.

There exist two standard methods for calculating loudness of (steady) broadband noise [35], a method described in the ANSI S3.4-2007 standard [36] and a method described in the DIN 45631:1991/ISO 532B standard [37,38].

The ANSI standard is based on a recently developed loudness model [39,40]. The standard can be used to calculate loudness from the spectrum of a sound signal, consisting of pure tones and/or noise bands such as 1/3-octave bands. The standard applies to binaural listening by persons with normal hearing in free field conditions (the method can also be used for monaural listening and diffuse field conditions, but this is not considered here; it was verified that the difference between free field and diffuse field results is of the order of 1 phon at most, in the cases considered in this article).

The DIN standard is based on a loudness model developed by Zwicker [41]. The standard can be used to calculate loudness from a 1/3-octave band spectrum for stationary sounds, for free field or diffuse field listening conditions. Loudness calculated with this standard is sometimes referred to as Zwicker loudness.

## 3. Analysis of Loudness Levels for Various Noise Spectra

This section presents an analysis of loudness levels calculated for various noise spectra. Pure tones are considered in Sections 3.1 and 3.2. Band-limited noise spectra are considered in Section 3.3. Broadband noise spectra that are representative for various types of environmental noise sources in practice are considered in Section 3.4.

### 3.1. Pure Tones—Equal Loudness Contours and Equal Sound Level Contours

**Figure 1.**
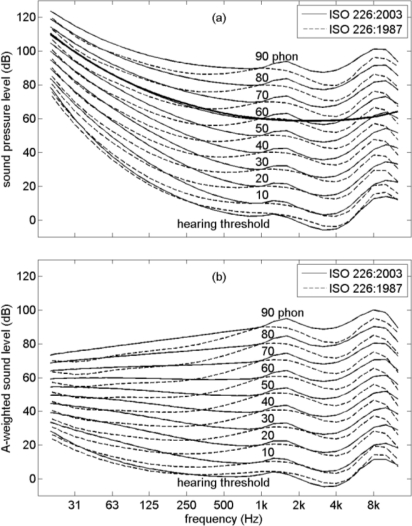
Equal-loudness contours and hearing threshold of pure tones, as a function of frequency and sound pressure level **(a)** and A-weighted sound level **(b)**, according to ISO 226:2003 and ISO 226:1987. The thick line represents the A-weighting curve.

Figure 1a shows equal-loudness contours of pure tones at 10, 20, …, 90 phon, as a function of sound pressure level and frequency, according to ISO 226:2003 and ISO 226:1987. The graph also shows the hearing threshold and the A-weighting curve (drawn through the point at 60 dB and 1 kHz). In the range from 20 Hz to 1 kHz, the ISO 226:2003 contours are considerably steeper than the ISO 226:1987 contours. The A-weighting curve closely follows the 60 phon ISO 226:2003 contour below 1 kHz.

Figure 1b shows the same contours as in Figure 1a, but now as a function of A-weighted sound level and frequency. The 60 phon ISO 226:2003 contour below 1 kHz is now a horizontal line in good approximation.

**Figure 2.**
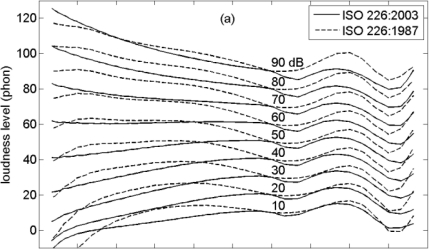

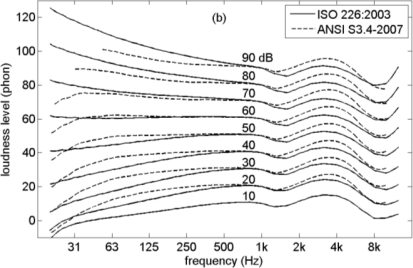
Contours of equal A-weighted sound level as a function of frequency and loudness level, according to ISO 226:2003 and ISO 226:1987 **(a)** and according to ISO 226:2003 and ANSI S3.4-2007 **(b)**.

Figure 2a shows the *inverse* contours of Figure 1b: contours of equal A-weighted sound level at 10, 20, …, 90 dB as a function of loudness level and frequency, according to ISO 226:2003 and ISO 226:1987. In this graph the 60 dB ISO 226:2003 contour below 1 kHz is a horizontal line in good approximation. Consequently, the A-weighted sound level is an accurate indicator of loudness for levels around 60 dB (below 1 kHz). Above 60 dB loudness slightly increases with decreasing frequency, and below 60 dB loudness slightly decreases with decreasing frequency, according to ISO 226:2003. The standard ISO 226:1987 yields a larger loudness increase with decreasing frequency in a large region of the frequency—A-weighted sound level diagram. This LF loudness increase has been described as an ‘underestimation by the A-weighting of LF loudness’ [10,11]. Since ISO 226:2003 yields a smaller loudness increase than ISO 226:1987 does, we included contours of both standards in Figures 1 and 2.

Figure 2b compares the ISO 226:2003 contours from Figure 2a with the corresponding contours according to the standard ANSI S3.4-2007. The ANSI standard more or less agrees with ISO 226:2003, in particular on the constant loudness of the 60 dB contour below 1 kHz, except below 31 Hz.

### 3.2. Pure Tones—Loudness as a Function of Sound Pressure Level

**Figure 3.**
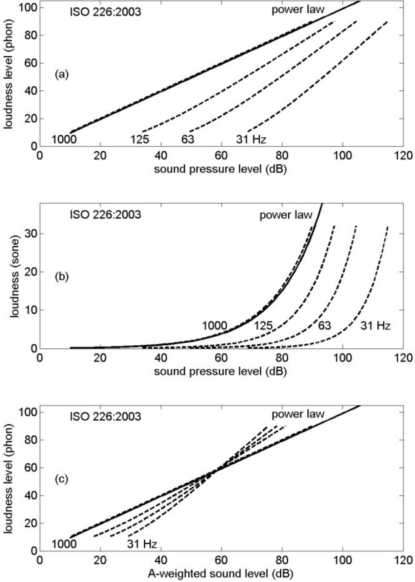
Loudness level **(a)** and loudness **(b)** as a function of sound pressure level, and loudness level as a function of A-weighted sound level **(c)**, for pure tones with frequencies 1000, 125, 63, and 31 Hz, according to ISO 226:2003 (dashed lines), and the 0.3 power law (solid line).

Figure 3a shows the loudness level as a function of sound pressure level, for pure tones with frequencies 1000, 125, 63, and 31 Hz, according to ISO 226:2003. Figure 3b shows the corresponding curves for loudness (*N*), which is related to loudness level (*L_N_*) by:

(1) $$N=2^{(L_{N}-40)/10}$$

so a loudness level change of 10 phon corresponds to a factor of 2 in loudness. The solid lines in Figures 3a and b represent the 0.3 power law for loudness [42]:

(2) $$N=K10^{0.3L_{p}/10}$$

where *L_p_* is the sound pressure level and *K* is a constant. Equations (1) and (2) yield the following expression for the loudness level as a function of sound pressure level:

(3) $$L_{N}=L_{p}\frac{0.3}{\text{lg}\;2}+40+\frac{10\;\text{lg}K}{\text{lg}\;2}$$

where ‘lg’ denotes logarithm to the base 10. Consequently, the power law in Figure 3a is a straight line. The slope is unity, since 0.3/lg2 = 1. The constant *K* has been chosen such that the loudness level for 60 dB is 60 phon, *i.e.*, the value for 1 kHz. This gives *K* = 0.063 (to distinguish the dashed and solid lines, *K* = 0.060 has been used for the graph). Consequently, the 0.3 power law corresponds to *L_N_* = *L_p_* for 1 kHz, in agreement with the definition of the loudness level of a tone as the sound pressure level of an equally loud 1 kHz tone.

The ISO 226:2003 loudness levels and loudness values in Figures 3a and 3b agree with the 0.3 power law for 1 kHz. For low frequency, deviations occur from the 0.3 power law: in Figure 3a the dashed lines are shifted to higher sound pressure levels and are also steeper. The shift disappears if A-weighting is applied to the sound pressure level, as shown in Figure 3c. The lines cross each other at the point at 60 phon and 60 dB. The slope at 31 Hz is about a factor of 2 larger than the slope at 1 kHz. This implies that a 0.6 power law holds at 31 Hz. These results are used in Section 3.4.

### 3.3. Band-Limited Noise

**Figure 4.**
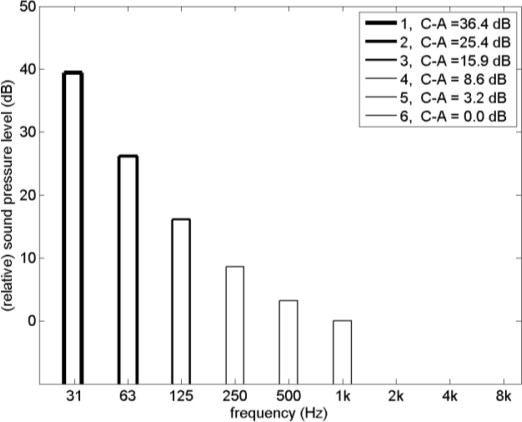
Six spectra with sound energy confined to a single 1/3-octave band. Values of the difference between the C-weighted sound level and the A-weighted sound level, denoted as C–A, are indicated in the legend.

**Figure 5.**
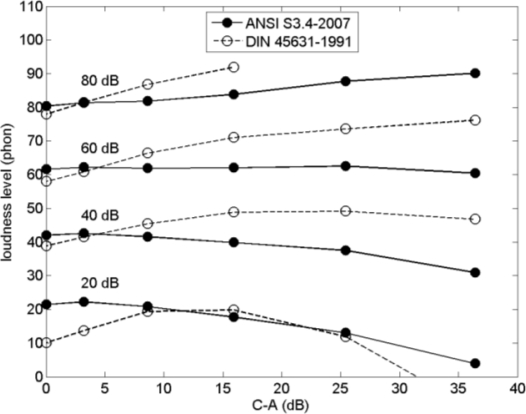
Loudness level as a function of C–A, calculated with ANSI S3.4-2007 and DIN 45631-1991 for the spectra shown in Figure 4, for A-weighted sound levels of 20, 40, 60, and 80 dB.

Figure 4 shows six spectra with sound energy confined to a single 1/3-octave band. Figure 5 shows loudness levels calculated for these spectra with ANSI S3.4-2007 and DIN 45631-1991, for A-weighted sound levels of 20, 40, 60, and 80 dB (the spectra shown in Figure 4 are normalized to zero A-weighted sound level). The loudness levels in Figure 5 are plotted as a function of the difference between the C-weighted sound level and the A-weighted sound level, denoted as C–A for simplicity. Quantity C–A is an indicator of the LF content of a spectrum [10,43], and the values of C–A for the six spectra are given in the legend of Figure 4. The ANSI curves in Figure 5 show that the loudness level is approximately constant at 60 dB, increases with C–A for 80 dB, and decreases with C–A for 20 and 40 dB. This corresponds directly to the behavior of loudness of pure tones as a function of frequency, shown in Figure 2b. The DIN curves in Figure 5 show a larger increase of loudness level with C–A, similar to the loudness level of pure tones according to ISO 226:1987, shown in Figure 2a.

In Section 2 it has been indicated that the standard ISO 226:2003 is an updated version of the older ISO 226:1987 standard. Since the ANSI S3.4-2007 standard agrees with ISO 226:2003 for pure tones, the ANSI standard has been used for the results presented in the remainder of this article for band-limited noise spectra and broadband noise spectra.

A spectrum of environmental noise usually covers a wide frequency range, rather than a single 1/3-octave band, so it is important to include the effect of bandwidth on loudness levels in this study. In the remainder of this section the bandwidth of a simple spectrum is varied in a systematic way, while in Section 3.4 more realistic broadband spectra are considered.

**Figure 6.**
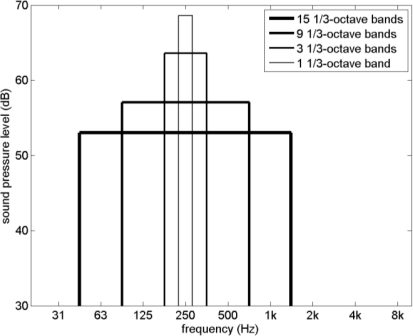
Four rectangular noise spectra, with acoustic energy confined to one, three, nine, and fifteen 1/3-octave bands.

**Figure 7.**
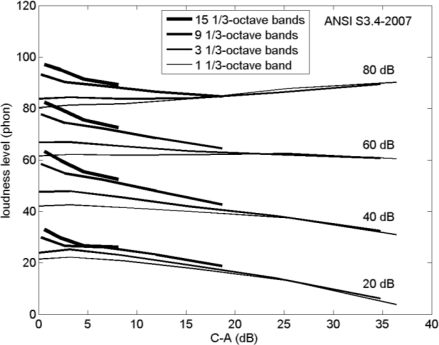
Loudness level as a function of C–A, calculated with ANSI S3.4-2007 for the spectra shown in Figure 6, for A-weighted sound levels of 20, 40, 60, and 80 dB, and center frequencies of 31, 63, 125, 250, 500, and 1000 Hz (see the text).

Figure 6 shows four band-limited noise spectra, corresponding to a rectangular band-pass filter covering one, three, nine, and fifteen 1/3-octave bands. Figure 7 shows the loudness level calculated for these spectra as a function of C–A, for A-weighted sound levels of 20, 40, 60, and 80 dB (Figure 6 is for 60 dB) and center frequencies of 31, 63, 125, 250, 500, and 1000 Hz (Figure 6 is for 250 Hz). The various combinations yield 21 different spectra.

Figure 7 shows that, at low values of C–A, the loudness level increases with increasing bandwidth. This is due to the effect of critical bands in human hearing (see Section 2). The loudness level is 15 to 20 phon higher for a spectrum of fifteen 1/3-octave bands than for a spectrum of a single 1/3-octave band. With increasing C–A, the loudness level increase with bandwidth becomes smaller. The loudness level increase is related to the number of critical bands covered by the spectrum, *i.e.*, the ratio of the spectrum bandwidth to the critical bandwidth. With decreasing frequency, 1/3-octave bands become narrower while the critical bandwidth levels off at 100 Hz below a frequency of 500 Hz. For C–A values larger than 15 dB the effect of increasing the bandwidth from one to three 1/3-octave bands is small (see Figure 7), which reflects the fact that a spectrum of three 1/3-octave bands is narrower than a critical band at low frequency. These results are used in Section 3.4.

### 3.4. Broadband Spectra

**Figure 8.**
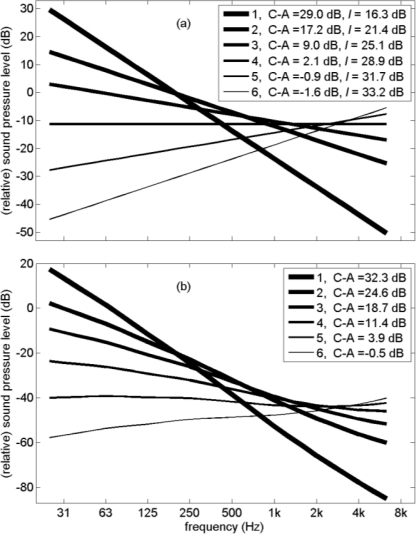
Six linear outdoor 1/3-octave band spectra normalized such that the A-weighted sound level is zero **(a)**, and corresponding indoor spectra normalized to zero *outdoor* A-weighted sound level **(b)**. Values of LF parameter C–A and façade insulation *I* are indicated in the legend.

Figure 8a shows six linear broadband noise spectra with gradients ranging from −10 dB/octave to +5 dB/octave. The spectra roughly cover the range of (average) gradients of broadband environmental noise spectra in practice [11,13,32,44], although the spectra are often not linear. Examples of approximately linear spectra are aircraft groundnoise spectra measured in communities near an airport [32], with a gradient of about −5 dB/octave, and the sound pressure level decreasing from 65−80 dB at 20 Hz to 20–30 dB at 8 kHz. Other environmental noise spectra are more or less linear up to a frequency of typically 1 kHz or 2 kHz, and fall off more steeply above this frequency. Of course, many environmental noise spectra have a more complex shape. Moreover, the shape of the spectrum changes with increasing distance from the noise source, due to frequency-dependent atmospheric propagation attenuations (see Section 2). Although the six spectra in Figure 8a do not represent the wide variety of spectrum shapes of environmental noise, they can be used to study the effect of LF content on the loudness of broadband environmental noise. Results of calculations for other spectra (not shown here), including spectra with a steep decay above 1 kHz and/or below 100 Hz, indicate that the conclusions about the effects of bandwidth and LF content on loudness presented in this section are valid for broadband environmental noise spectra in general. It should be noted that we ignore the effect of background noise, which may affect the loudness levels.

The LF content of the spectra shown in Figure 8a is represented by parameter C–A. The values of C–A are indicated in the legend, and range from −1.6 dB to 29 dB. In practice, the value of C–A for road traffic noise is typically between zero and 18 dB [13]. For other noise sources, such as some LF industrial sources, larger values of C–A occur. Unpublished results of outdoor measurements performed by the Dutch consultancy firm Peutz, reported in 2003 to the Dutch Ministry of Environment, yielded the following ranges of C–A: 2–15 dB for road traffic noise, 1–15 dB for rail traffic noise, 9–21 dB for ship noise, 2–13 dB for aircraft noise (not including aircraft groundnoise), and 6–24 dB for various types of industrial noise. Indoor measurements resulted in larger values of C–A. Recently reported aircraft groundnoise spectra [32], measured outdoors in a community at 2 to 3 km from the fifth runway of Schiphol airport for B737, B747, and MD11 aircraft departures, yield values of C–A in the range 14–22 dB.

**Figure 9.**
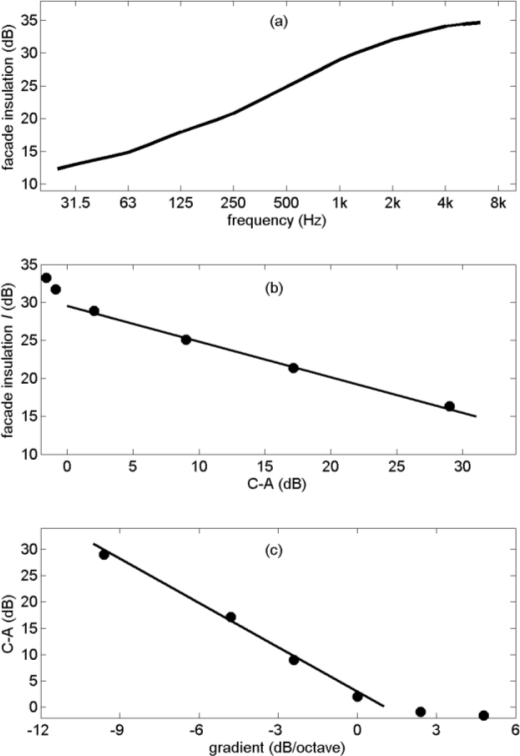
Spectrum of façade insulation used in this study [16] **(a)**, A-weighted façade insulation *I* as a function of C–A **(b)**, and value of C–A as a function of the gradient of the linear spectrum **(c)**, for the six spectra shown in Figure 8a. The straight lines are guides to the eye.

The spectra shown in Figure 8a are assumed to represent *outdoor* noise, for example noise at the façade of a house, originating from outdoor noise sources such as traffic and industrial sources. Figure 8b shows corresponding *indoor* spectra, calculated with the façade insulation spectrum shown in Figure 9a. This façade insulation spectrum should only be considered as a typical representative example, as insulation spectra in practice show large variations. The increase of façade insulation with frequency is a typical characteristic of façade insulation spectra.

The strong frequency dependence of façade insulation can be understood from the expression for the transmission loss *R* for a solid wall (and normal sound wave incidence):

(4) $$R(\omega )=10\text{lg}\left[1+{\left(\frac{\omega m}{2Z}\right)}^{2}\right]$$

which is commonly referred to as the mass law for sound transmission [28,45,46]. Here, *m* is the mass of the wall per unit area, *Z* is the impedance of air, and *ω* is the angular frequency of the transmitted sound wave. For *ω* >> 2*Z*/*m* the transmission loss increases by 6 dB per octave. For example, for *m* = 50 kg/m^2^, the expression yields a transmission loss of 12 dB at 10 Hz and about 50 dB at 1 kHz. Sound transmission through the façade of a house depends not only on the walls, but also on various structural elements and openings in the façade (air ventilation, windows, sealing), which limit the façade insulation spectrum at high frequency. Consequently, façade insulation spectra in practice show large variations [47–52], depending on the wide variety of façade structures of houses. Moreover, opening and closing of windows of houses plays an important role in the variations of façade insulation.

Values of the broadband A-weighted façade insulation, denoted with the symbol *I*, are indicated in the legend of Figure 8a. With increasing C–A, façade insulation *I* decreases. This is shown graphically in Figure 9b. Façade insulation *I* decreases linearly with C–A, except for negative values of C–A. Figure 9c shows how C–A varies with the gradient of the linear spectrum. Again the behavior is approximately linear for positive values of C–A, corresponding to negative gradients. The linear behavior in Figures 9b and 9c was verified also for other negative gradients than those represented in Figure 8a.

**Figure 10.**
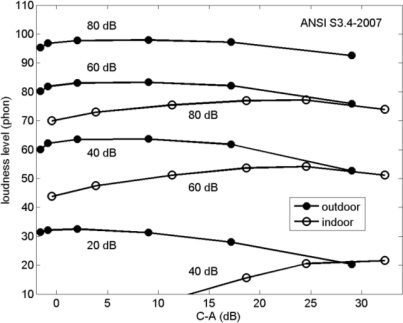
Loudness level as a function of C–A calculated with ANSI S3.4-2007 for the spectra shown in Figures 8a and 8b, for outdoor A-weighted sound levels of 20, 40, 60, and 80 dB.

Figure 10 shows loudness levels calculated with the ANSI S3.4-2007 standard for the spectra shown in Figures 8a and 8b, for A-weighted sound levels of 20, 40, 60, and 80 dB. These values of the A-weighted sound level represent *outdoor* noise: the spectrum shapes shown in Figures 8a and 8b have been shifted such that they correspond to *outdoor* A-weighted sound levels of 20, 40, 60, and 80 dB. *Only* in this way the increase of indoor loudness with increasing LF parameter C–A, at constant outdoor A-weighted noise level, is taken into account. It should be noted that the indoor values of C–A were calculated for the indoor spectra (to show the enhanced indoor LF content), but the corresponding outdoor values of C–A follow from the legends of Figures 8a and 8b. It should also be noted that high values of C–A are often associated with low sound levels at large distance from a noise source, due to the frequency-dependent atmospheric propagation attenuations mentioned in Section 2.

Figure 10 shows that the outdoor loudness level is constant or decreases with increasing C–A, except for values of C–A below 2 dB. The indoor loudness level, however, increases with increasing C–A, except for C–A above 25 dB and A-weighted sound levels of 60 and 80 dB. This behavior can be interpreted in terms of three effects.

First, the loudness level of pure tones or single 1/3-octave bands increases with C–A for A-weighted sound levels above 60 dB and decreases with C–A below 60 dB (see Sections 3.1−3.3). Second, an increase of the bandwidth of the spectrum causes an increase of the loudness level (see Section 3.3), but since the increase is larger at small values of C–A than at large values, the effect of a bandwidth increase is also a decrease of the slopes of the curves (see Figure 7). This explains why the slopes of the outdoor curves in Figure 10 are smaller than the slopes for pure tones and single 1/3-octave bands. The third effect that plays a role is the effect of façade insulation. Façade insulation decreases with increasing C–A (see Figure 9b), and this causes an increase of *indoor* loudness level with C–A at constant *outdoor* A-weighted sound level. The slopes of the indoor curves in Figure 10 are considerably larger than the slopes of the outdoor curves, which indicates that the effect of façade insulation on indoor loudness is an important effect.

**Figure 11.**
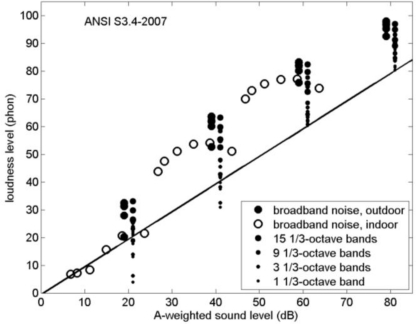
Loudness levels from Figure 10 and from Section 3.3 as a function of A-weighted sound level. The straight line represents the 0.3 power law at 1 kHz. For clarity, filled symbols have been shifted by +1dB or −1dB from 20, 40, 60, and 80 dB.

Figure 11 shows the loudness levels from Figure 10 as a function of A-weighted sound level rather than C–A. For the indoor loudness levels, indoor A-weighted sound levels were used here. Also included in the graph are the loudness levels from Section 3.3 for band-limited noise. The straight line represents the 0.3 power law at 1 kHz. The graph illustrates the loudness level increase due to a bandwidth increase, but also the change from the 0.3 power law behavior for 1 kHz tones or single 1/3-octave bands to a 0.6 power law behavior for LF tones or single 1/3-octave bands (by the dots below the straight line in Figure 11; *cf.* Figure 3c). For broadband environmental noise the graph shows loudness level variations of the order of 10 phon, at constant A-weighted sound level.

**Figure 12.**
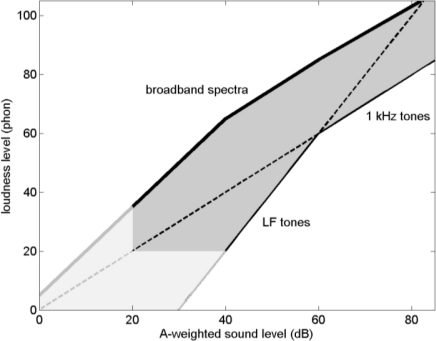
Schematic representation of Figure 11. The gray area represents the range of loudness levels of noise spectra in practice, and is bounded by the 0.3 power law for 1 kHz tones, the 0.6 power law for LF tones, and an upper curve for broadband noise.

Figure 12 shows the graph in Figure 11 in a more schematic way. The gray area represents loudness levels of noise spectra considered in this article, both the spectra shown in Figures 8a and 8b and the band-limited spectra considered in Section 3.3. The gray area is bounded by the 0.3 power law for 1 kHz tones or single 1/3-octave bands, the 0.6 power law for LF tones or single 1/3-octave bands (both power laws go through the point at 60 phon and 60 dB; see Section 3.2), and a curve that represents the highest loudness levels in Figure 11 [The curve is approximated by straight segments through the points (0,5), (20,35), (40,65), (60,85) and (82,105)].

Loudness levels calculated for other broadband noise spectra (not shown here), including the spectra shown in Figures 8a and 8b with an additional decay of 15 dB per octave above 1 kHz and/or below 100 Hz, also fall in the gray area in Figure 12. The effect of introducing the additional decays is a change of loudness level of ±6 phon at most. The change is negative for points near the upper boundary of the gray area, so the points stay within the gray area. It is concluded that the gray area covers loudness levels of environmental noise spectra that occur in practice. Levels of broadband spectra are concentrated in the upper part of the gray area (see Figure 11).

The area below 20 dB and 20 phon in the graph is shown in light gray, for two reasons: (i) sound levels below 20 dB are not relevant for most practical situations, and (ii) the accuracy of loudness calculation standards is limited below 20 phon.

## 4. Conclusions

The effects of bandwidth and LF content on the loudness level of environmental noise have been analyzed in this article. In Figure 12 it has been shown that the upper limits of the loudness level for broadband environmental noise spectra are about 20 to 40 phon higher than the lower limits for pure tones (ignoring the region below 20 phon in the graph).

The effect of bandwidth on the loudness level at constant A-weighted sound level has been shown in Figure 11. For broadband environmental noise, loudness level variations of the order of 10 phon occur.

The effect of LF content on the loudness level of broadband environmental noise has been shown in Figure 10. The variation of the loudness level with LF content depends on the shape of the spectrum, which is different for outdoor noise and indoor noise due to the frequency dependence of façade insulation. The outdoor loudness level remains constant or decreases with increasing LF parameter C–A, except for values of C–A below 2 dB. The indoor loudness level, however, increases with increasing C–A in a large part of the diagram.

The analysis presented here may be used as a starting point for assessing limitations and potential improvements of environmental noise control methods based on A-weighted sound levels or loudness levels. A possible approach is to apply specific weightings, or penalties, for certain acoustic parameters, such as LF content, bandwidth, and tonality. Ultimately, such weightings or penalties should be based on noise annoyance studies, but considering the effect of the acoustic parameters on loudness may be helpful, since loudness is related to annoyance but is a less complex quantity than annoyance is.
